# A Structural Model of the Pore-Forming Region of the Skeletal Muscle Ryanodine Receptor (RyR1)

**DOI:** 10.1371/journal.pcbi.1000367

**Published:** 2009-04-24

**Authors:** Srinivas Ramachandran, Adrian W. R. Serohijos, Le Xu, Gerhard Meissner, Nikolay V. Dokholyan

**Affiliations:** 1 Department of Biochemistry and Biophysics, University of North Carolina Chapel Hill, Chapel Hill, North Carolina, United States of America; 2 Molecular and Cellular Biophysics Program, University of North Carolina Chapel Hill, Chapel Hill, North Carolina, United States of America; 3 Department of Physics and Astronomy, University of North Carolina Chapel Hill, Chapel Hill, North Carolina, United States of America; Stanford University, United States of America

## Abstract

Ryanodine receptors (RyRs) are ion channels that regulate muscle contraction by releasing calcium ions from intracellular stores into the cytoplasm. Mutations in skeletal muscle RyR (RyR1) give rise to congenital diseases such as central core disease. The absence of high-resolution structures of RyR1 has limited our understanding of channel function and disease mechanisms at the molecular level. Here, we report a structural model of the pore-forming region of RyR1. Molecular dynamics simulations show high ion binding to putative pore residues D4899, E4900, D4938, and D4945, which are experimentally known to be critical for channel conductance and selectivity. We also observe preferential localization of Ca^2+^ over K^+^ in the selectivity filter of RyR1. Simulations of RyR1-D4899Q mutant show a loss of preference to Ca^2+^ in the selectivity filter as seen experimentally. Electrophysiological experiments on a central core disease mutant, RyR1-G4898R, show constitutively open channels that conduct K^+^ but not Ca^2+^. Our simulations with G4898R likewise show a decrease in the preference of Ca^2+^ over K^+^ in the selectivity filter. Together, the computational and experimental results shed light on ion conductance and selectivity of RyR1 at an atomistic level.

## Author Summary

Ryanodine receptors (RyRs) are ion channels present in the membranes of an intracellular calcium storage organelle, the sarcoplasmic reticulum. Nerve impulse triggers the opening of RyR channels, thus increasing the cytoplasmic calcium levels, which subsequently leads to muscle contraction. Congenital mutations in a specific type of RyR that is present in skeletal muscles, RyR1, lead to central core disease (CCD), which leads to weakened muscle. RyR1 mutations also render patients to be highly susceptible to malignant hyperthermia, an adverse reaction to general anesthesia. Although it is generally known that CCD mutations abort RyR1 function, the molecular basis of RyR1 dysfunction remains largely unknown because of the lack of atomic-level structure. Here, we present a structural model of the RyR1 pore region, where many of the CCD mutations are located. Molecular dynamics simulations of the pore region confirm the positions of residues experimentally known to be relevant for function. Furthermore, electrophysiological experiments and simulations shed light on the loss of function of CCD mutant channels. The combined theoretical and experimental studies on RyR1 elucidate the ion conduction pathway of RyR1 and a potential molecular origin of muscle diseases.

## Introduction

Muscle contraction upon excitation by nerve impulse is initiated by a rapid rise in cytoplasmic Ca^2+^. In skeletal muscle, the rise in cytoplasmic Ca^2+^ is brought about by the opening of the ryanodine receptor (RyR1), which releases Ca^2+^ from intracellular stores [1],[2]. RyRs are large tetrameric ion channels (molecular weight of 2.26 MDa) present in the membranes of endoplasmic/sarcoplasmic reticulum. They have high conductance for monovalent (∼800 pS with 250 mM K^+^ as conducting ion) and divalent cations (∼150 pS with 50 mM Ca^2+^), while being selective for divalent cations (*P*_Ca_/*P*_K_∼7) [3]. RyRs are important mediators of excitation-contraction coupling and congenital mutations of RyRs result in neuromuscular diseases such as malignant hypothermia and central core disease (CCD) [4].

Although RyRs are physiologically important, the molecular basis of their function is poorly understood. RyRs have unique properties such as their modes of selectivity and permeation not seen in other ion channels with known structures. Next to the putative selectivity filter (4894GGGIG), there are two negatively charged residues (D4899 and E4900) in RyR1 that are essential for normal selectivity and conductance [5]. K^+^ channels have an analogous selectivity filter, but in contrast to RyR1, have only one adjacent negative residue that is not even conserved while other Ca^2+^ channels have only one conserved negative residue in the equivalent position [6]. In the selectivity filter, mutations result in non-functional channels [4] leading to CCD. A structural model of the pore region that would reveal the location and function of these residues will be useful in understanding the role of these residues in channel function.

An early model of RyR ion permeation postulated potential barriers within the pore corresponding to three putative binding sites [7]. Without any knowledge of the structure of the pore, the model was able to quantitatively reproduce conductance data of various ions. A PNP-DFT (Poisson Nernst Planck-Density Functional Theory) model [8] accurately modeled the role of residues D4899 and E4900 in RyR1 in generating the high ion conductances of RyRs established by mutagenesis [5],[9]. Selectivity was attributed to charge-space competition, as Ca^2+^ could accommodate the most charge in least space compared to K^+^. However, since the channel model used in these simulations relied on a fixed structure, it could not predict changes due to mutations that potentially alter the structure of the channel.

Additionally, there are two homology models of the RyR pore region [10],[11] based on KcsA, a bacterial K^+^ channel whose solution structure is known [12]. Shah *et al.* [11] used bioinformatics approaches to construct models for RyR and the related inositol triphosphate channel. The luminal loop in their RyR model begins at 4890G resulting in the selectivity filter being 4890GVRAGG. However, mutagenesis has shown that residues I4897, G4898, D4899 and E4900 are important for channel conductance and selectivity, which suggests that they are part of the conduction pathway of RyR1 resulting in the predicted selectivity filter being 4894GGGIGDE. Welch *et al.* also constructed a homology model for the cardiac ryanodine receptor (RyR2) using the structure of the KcsA channel [10] and performed simulations to identify residues important for channel function. Their simulations failed to identify D4899 as an important residue for ion permeation contrary to what has been shown experimentally [5]. Furthermore, cryo-electron microscopy (cryo-EM) of RyR1 (which has revealed the pore structure at highest resolution yet) revealed significant differences between the pore region of KcsA and RyR1 [13].

Experimental structure determinations of the RyRs have been mainly performed by cryo-EM [14]–[17]. These studies revealed conformational changes that accompany channel opening [18] and the binding sites of various effectors of RyRs [19]–[21]. Cryo-EM has a resolution of ∼10–25 Å and thus is able to provide only limited structural information regarding the pore structure. Samso *et al.* [22] manually docked the KcsA pore structure into the transmembrane region of their cryoEM map of the intact closed RyR1. Furthermore, they predicted the presence of at least 6 transmembrane helices from simple volumetric constraints. Ludtke *et al.* [13] identified several secondary structure elements in their ∼10 Å resolution cryo-EM map of the closed RyR1. The pore-forming region as visualized by Ludtke *et al.* consists of a long inner helix made up of 31 residues and a pore helix made up of 15 residues that are presumably connected by a long luminal loop made up of 24 residues. Since the structure is derived from cryo-EM, the positions of pore residues' side chains and the structure of loops connecting the helices are unknown. We build a molecular model of the pore region of RyR1 based on their cryo-EM study by adding the luminal loop and the missing side chains of residues forming the helices of the pore. Furthermore, in our molecular dynamics simulations we examine the interactions of the pore region with mono- and divalent cations known to permeate the channel (Table 1).

**Table 1:** Concentrations of ions used in different simulations.

Simulation	KCl (mM)	NaCl (mM)	CaCl_2_ (mM)	Total Simulation Length (ns)
RyR1-WT CaCl_2_	-	-	125	90
RyR1-WT KCl	250	-	-	90
RyR1-WT CaCl_2_/KCl	250	-	70	90
RyR1-WT NaCl/KCl	250	70	-	15
RyR1-D4899Q CaCl_2_/KCl	250	-	70	90
RyR1-D4899Q KCl	250	-	-	90
RyR1-G4898R CaCl_2_/KCl	250	-	70	84
RyR1-G4898R KCl	250	-	-	90

## Results

### Model of the RyR1 pore region

**Figure 1:**
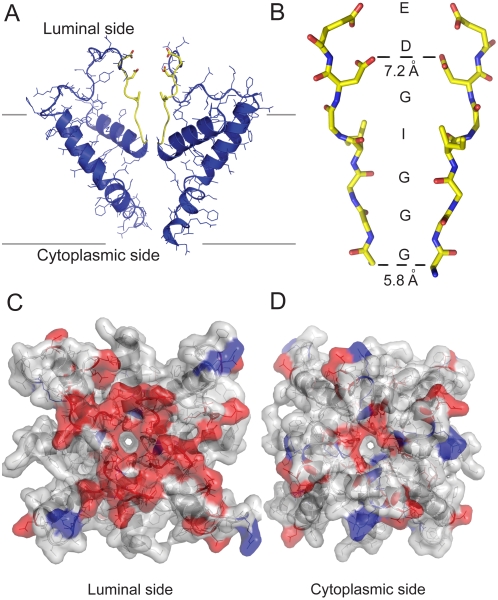
The pore model. (A) Two opposing RyR1 monomers shown in cartoon representation. The selectivity filter (the motif 4894GGGIGDE) is shown in yellow. (B) Selectivity filter region of two opposing RyR1 monomers shown in stick representation. The sequence of the fragment is shown along the path of the ions. The molecular surface of the luminal face (C) and the cytoplasmic face (D) of RyR1 pore tetramer. Negative residues are shown in red while positive residues are shown in blue and the neutral residues are shown in white. This figure was created using PyMOL (http://pymol.sourceforge.net/).

We present in Figure 1 an atomistic model of the pore-forming region of the tetrameric RyR1 constructed from cryo-EM data [13]. Figure 1A shows two of the four inner helices and pore helices connected by a long luminal loop. Site-directed mutagenesis [22],[23] predicts that RyR1 has a selectivity filter (4894GGGIGD), which is analogous to K^+^ channels (Figure 1B). Since the pore helix immediately precedes the selectivity filter, we assign M4879-A4893 to the 15-residue pore helix. In K^+^ channels, the sequence GXXXXA in the inner membrane-spanning helix has been proposed to form the gating hinge [24]. The analogous glycine in RyR1 occurs in the 4934 position, which determines the sequence of the 40-residue inner helix as I4918-E4948. Thus, the pore corresponds to residues M4879-E4948 [13], which includes the putative selectivity filter. We construct the luminal loop by constraining the diameter of the selectivity filter at its luminal edge to 7 Å [25] (Figure 1B). In this model, the acidic residues important for channel function, D4899 and E4900 are located at the mouth of the pore and at the beginning of the selectivity filter.

Both the cytoplasmic and luminal faces of the pore are highly negatively charged (as seen in the surface representation in Figure 1C and 1D), which may allow cations to concentrate around the mouths of the pore. We also predict the negatively charged faces to exclude anions, which RyR1 is known not to conduct. Interestingly, with the exception of L4935 and L4943, the hydrophobic residues lining the helices face away from the water filled pore. In K^+^ channels, hydrophobic residues facing the pore are known to perform important functional roles (like stabilization of the inactivation gate [26]). The model exhibits structural similarities with the K^+^ channel MthK such as the positioning of the pore helix and the inner helix and the bending of the inner helix [27], although in RyR1 the pore is significantly wider. To model the interactions of RyR1 pore with ions and elucidate sites of high ion occupancy along the pore, we perform molecular dynamics simulations of RyR1 (see below).

### Experimental characterization of wild type and mutant channels

**Figure 2:**
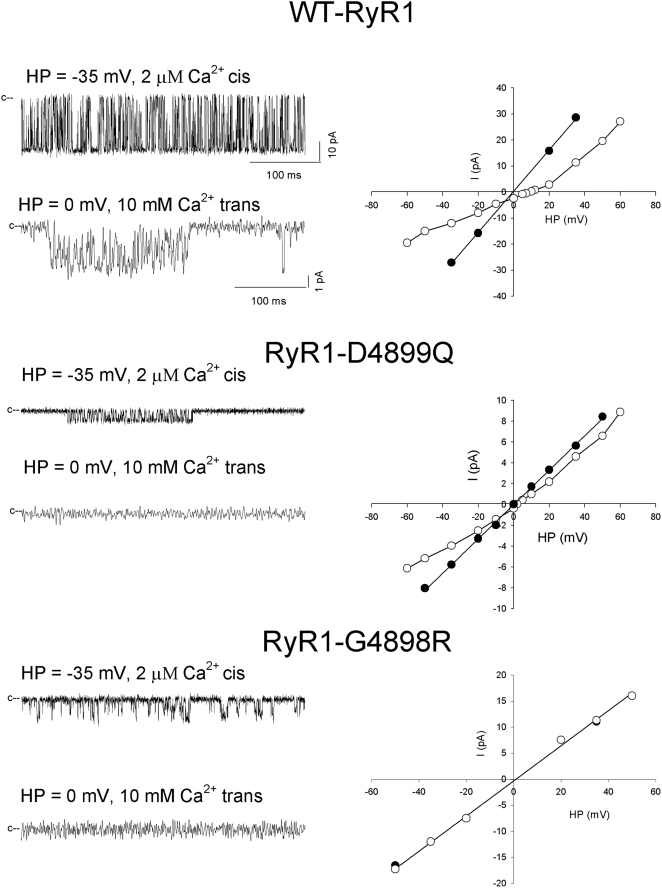
Ion permeation properties of RyR1-WT (top), RyR1-D4899Q (middle), and RyR1-G4898R (bottom) ion channels. Upper traces represent single channel currents (openings shown as downward deflections from the closed state (c–) recorded in symmetrical 250 mM KCl and 5 µM free Ca^2+^ cis. Bottom traces represent single channel currents recorded at 0 mV following the addition of 10 mM trans Ca^2+^.

**Figure 3:**
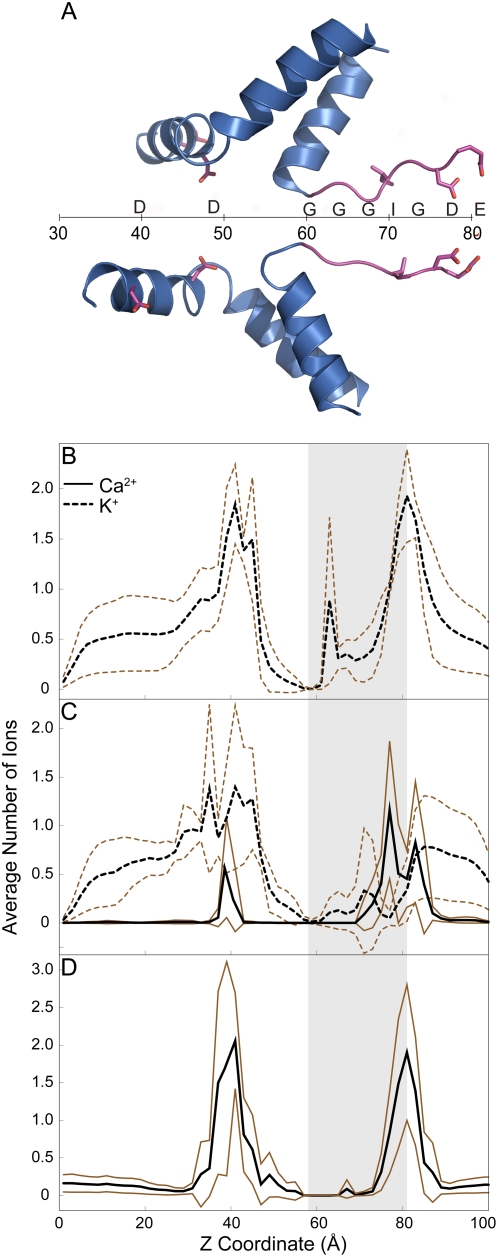
Histogram of ion occupancies along the axis of the channel. (A) Schematic of the pore axis and the positions of residues along the pore axis. (B) RyR1-WT in 250 mM KCl. (C) RyR1-WT in 250 mM KCl and 70 mM CaCl_2_. (D) RyR1-WT with 125 mM CaCl_2_. The region corresponding to selectivity filter is shaded in the plots. This region was determined from the distribution of z coordinates of Cα of G4894 and the carboxyl group of E4900 from each of the simulations. The average occupancy from 4 simulations is plotted in black, while lines corresponding to ±1 SD are plotted in brown.

We describe here some of the experimental characteristics of RyR1 mutants D4899Q and G4898R, which are both present in the selectivity filter. We compare in Figure 2 the ion permeation properties of wild type RyR1-WT [5] with RyR1-D4899Q [5] and CCD associated RyR1-G4898R [28] mutant channels. Proteoliposomes containing purified 30S channel complexes were fused with planar lipid bilayers and single channel currents were recorded in 250 mM KCl on both sides of the bilayer. Figure 2 shows representative single channel traces in presence of 2 µM cis (SR cytosolic) Ca^2+^ (left, upper traces) and following the subsequent addition of 10 mM trans (SR luminal) Ca^2+^ (left, lower traces). In presence of 2 µM Ca^2+^, WT and the two mutant channels showed rapid transitions between open and closed channel states. Reduction in cis Ca^2+^ from 2 µM to 0.01 µM reduced channel open probability for WT and D4899Q close to background levels (data not shown). In contrast, single channel activities of RyR1-G4898R did not respond to a change in cytosolic Ca^2+^ concentration from 2 µM to 0.01 µM (not shown). Ion currents through WT and mutant channels showed a linear voltage-dependence but differed in their magnitude (Figure 3, right panel). Averaged single channel conductances were 801 pS for WT, 164 pS for RyR1-D4899Q, and 352 pS for RyR1-G4898R. The Ca^2+^ selectivity of WT and the two RyR1 mutants was determined by recording current-voltage curves in 250 mM symmetrical KCl with 10 mM Ca^2+^ in the trans bilayer chamber. At 0 mV in presence of 10 mM trans Ca^2+^, WT exhibited averaged unitary Ca^2+^ current of −2.4 pA compared with −0.4 pA for RyR1-D4899Q and ∼0 pA for RyR1-G4898R. Addition of 10 mM Ca^2+^ to the trans chamber reduced single channel currents of WT and D4899Q at negative and positive potentials and the averaged reversal potentials (E_rev_) for WT and D4899Q were shifted by +9.5 mV and +1.9 mV, respectively (Figure 2, right panel). Applying constant field theory, a permeability ratio of Ca^2+^ over K^+^ (P_Ca_/P_K_) of 7.0 and 1.0 is calculated for WT and D4899Q, respectively (Table 2). In contrast, addition of 10 mM trans Ca^2+^ did not generate a noticeable unitary Ca^2+^ current at 0 mV, and had no effect on ion currents or reversal potential of RyR1-G4898R. Taken together, the single channel data of Figure 2 indicate that the D4899Q mutation decreases K^+^ conductance and ion selectivity for Ca^2+^ over K^+^ compared to WT, without eliminating Ca^2+^ responsiveness. In contrast, the CCD associated G4898R mutation abolished Ca^2+^ responsiveness, Ca^2+^ permeation, and reduced ion conductance demonstrating that the G4898R mutation introduced major global conformational changes in RyR1.

**Table 2:** Experimental characteristics of RyR1-WT, RyR1-G4898R and RyR1-D4899Q.

	K^+^ Conductivity (pS)^1^	I_Ca_(pA)^2^	P_Ca/K_ 3	Caffeine^4^	Ryanodine^5^
Wild Type	801	−2.8	7	+	+
D4899Q	164	−0.4	1	+	+
G4898R	197	<0.1	−	−	−

1 K^+^ conductivity in pS with 250 mM KCl.

2 Ca^2+^ current recorded at 0 mV in symmetrical 250 mM KCl, 5 µM free Ca^2+^ cis and 10 mM trans Ca^2+^.

3 Selectivity of Ca^2+^ over K^+^.

4 Ca^2+^ release in response to caffeine.

5 Ca^2+^ release in response to ryanodine binding.

Acidic residues lining the pore of the RyR channel have been assumed to be deprotonated at physiological pH. In support of this, site directed mutagenesis resulting in charge neutralization of acidic residues reduced ion conduction and selectivity [5]. Single channel experiments in the pH range of 6.5–9.4 (on the luminal side) on RyR1-WT were performed to probe the protonation status of luminal residues D4899 and E4900. Lack of a significant effect of a change of pH on K^+^ ion conductance and the permeability ratio of Ca^2+^ over K^+^ (Xu and Meissner, unpublished studies) indicate that the protonation status of D4899 and E4900 remained unchanged. Hence we assume in our MD simulations that these residues are deprotonated.

### Molecular dynamics simulations of the pore model

To model the interactions of RyR1 pore with ions, we perform molecular dynamics simulations of the RyR1 pore tetramer with 70 mM CaCl_2_ and 250 mM KCl. Ca^2+^ is present in the solution only on the luminal side. We plot the histogram of ion occupancies in the pore against the pore axis in Figure 3. Ca^2+^ shows highest occupancy at 77 Å along the axis of the channel, which corresponds to the position of D4899 in the selectivity filter (Figure 3C). We find the preferential occupancy ratio, R for the CaCl/KCl simulations to be 11.3±5.6 (Table 3). These results show a clear preference of Ca^2+^ over K^+^ in the selectivity filter. We hypothesize that this preferential localization of Ca^2+^ in the first binding site along the path from luminal to cytosolic side may play an important role in channel selectivity. To ensure that this preferential localization of Ca^2+^ is not due to selective exclusion of K^+^ ions in the filter, we perform simulations of the pore with only KCl present. These simulations show K^+^ occupancy to be highest at 81 Å (Figure 3B) and the total charge of the ions in the selectivity filter (Table 3) to be similar to simulations with CaCl/KCl, which shows that the binding sites are amenable to K^+^ in absence of Ca^2+^. As a control, we perform simulations with NaCl/KCl with the same starting configuration as the CaCl_2_/KCl simulations. We observe a decreased occupancy of Na^+^ when compared to Ca^2+^. Na^+^ is still preferred over K^+^ in the selectivity filter, if we take relative concentration of NaCl and KCl into consideration with R = 2.2 (Figure S1). Simulations with only CaCl_2_ show similar results (Figure 3D). The occupancy data qualitatively reflects the selectivity of RyR1 pore, which is in the order of Ca^2+^ being much greater than Na^+^ which is slightly greater than K^+^ [7].

**Table 3:** Ion occupancy in the selectivity filter in different simulations.

Simulation	Number of ions^1^			Total number of ions in the selectivity filter	R‡
	Ca^2+^	K^+^	Na^+^		
RyR1-WT CaCl_2_	3.68±1.15	-	-	3.68±1.15	-
RyR1-WT KCl	-	6.94±1.08	-	6.94±1.08	-
RyR1-WT CaCl_2_/KCl	2.94±0.45	1.13±0.58	-	4.07±0.95	11.31±5.63
RyR1-WT NaCl/KCl	-	3.2	2	5.2	2.2
RyR1-D4899Q CaCl_2_/KCl	1.31±0.31	1.67±0.74	-	2.97±0.68	3.11±1.62
RyR1-D4899Q KCl	-	2.97±0.4	-	2.97±0.4	-
RyR1-G4898R CaCl_2_/KCl	2.26±0.15	1.75±0.27	-	4.01±0.22	4.48±0.9
RyR1-G4898R KCl	-	4.67±0.95	-	4.67±0.95	-

**Figure 4:**
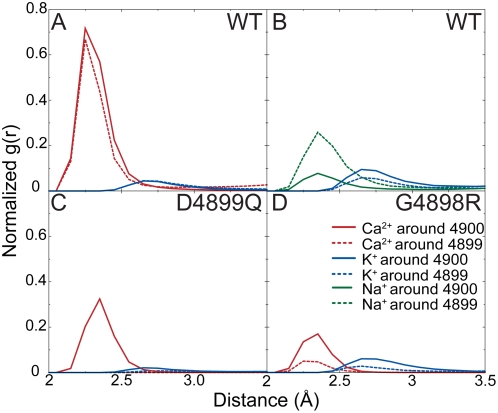
Radial distribution function (RDF) of ions around the carbonyl oxygens of 4899 and 4900. (A) RyR1-WT in 250 mM KCl and 70 mM CaCl_2_. (B) RyR1-WT in 250 mM KCl and 70 mM NaCl. (C) RyR1-D4899Q in 250 mM KCl and 70 mM CaCl_2_. (D) RyR1-G4898R in 250 mM KCl and 70 mM CaCl_2_. The plots represent distribution of the number of ions at a distance r from the centre of the carboxyl groups (amide group in Q) of D4899 and E4900. The graph axis starts at 2 Å and the peak occurs at the sum of van der Waal's radii of oxygen and the ion. The average occupancy from 4 simulations is plotted.

**Figure 5:**
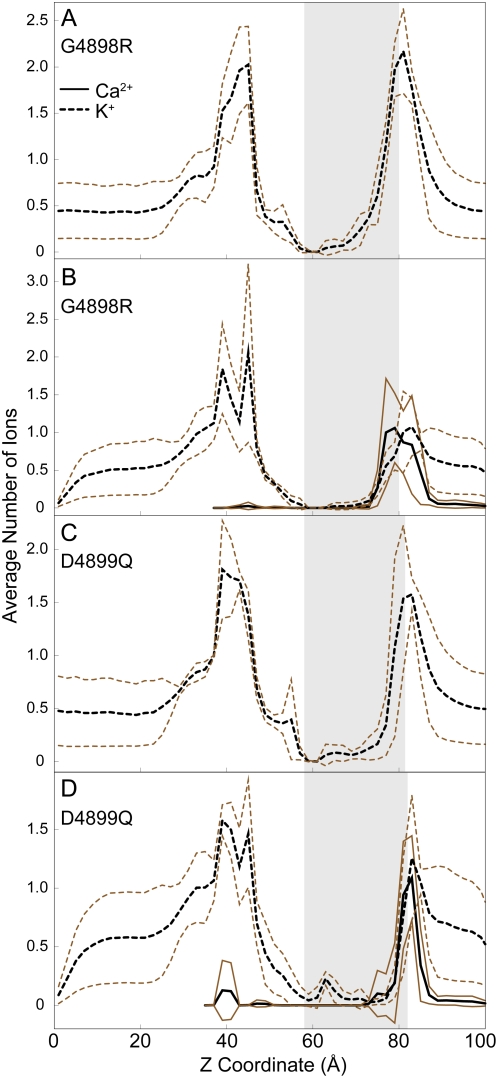
Histogram of ion occupancies along the axis of the mutant channels. (A) RyR1-G4898R in 250 mM KCl. (B) RyR1-G4898R in 250 mM KCl and 70 mM CaCl_2_. (C) RyR1-D4899Q in 250 mM KCl. (D) RyR1-D4899Q in 250 mM KCl and 70 mM CaCl_2_. The shaded region in the plots corresponds to the selectivity filter, determined the same way as in Figure 4. The average occupancy from 4 simulations is plotted in black, while lines corresponding to ±1 SD are plotted in brown.

1 In the selectivity filter, which is the shaded region shown in Figure 4 and Figure 5.

**‡:** Ratio of preferential occupancy of ion1 over ion2 normalized by total number of ions present in each species.

Radial distribution functions (RDF) of the ions around the carboxyl oxygens of D4899 and E4900 reflect the affinities of various ions to the binding site. We plot the number of ions found at a distance *r* from the carboxyl oxygens of D4899 and E4900, to confirm that the peaks that we observe in ion occupancy plots is due to localization of the ions near these residues. From the RDFs, we find that Ca^2+^ ions exhibit the highest affinity for D4899 and E4900 followed by Na^+^ and then K^+^ (Figure 4A and 4B). It is experimentally observed that the conductance of the channel is highest for K^+^, followed by Na^+^ and then Ca^2+^ [7]. These two observations lead us to postulate that that tighter binding results in ions spending more time in the channel, thus giving rise to lower ion currents.

The higher occupancy of Ca^2+^ compared to K^+^ can be either primarily attributed to electrostatics (due to the presence of 8 acidic residues in the vicinity) or to electrostatics combined with the structure formed by the selectivity filter. By calculating the occupancy of ions inside the pore region corresponding to the selectivity filter (formed by 4894GGGIGDE of the tetramer, as shown in Figure 1B), we also consider if the pore structure contributes in concentrating the ions inside the pore. Although eight negative charges in a confined space would be more selective for Ca^2+^ than K^+^ as determined by a charge/space model [29], we find that in an all-atom model of RyR1 pore region, the selectivity filter is able to sample structures that support preferential localization of Ca^2+^ inside the pore (in the region corresponding to the selectivity filter). Thus, the preferential localization of Ca^2+^ over K^+^ is due to both the pore structure and electrostatics.

### Molecular dynamics simulations of RyR1-G4898R and RyR1-D4899Q

To confirm the preferential Ca^2+^ binding in the selectivity filter, we perform MD simulations on two experimentally characterized RyR1 mutants, D4899Q [5] and G4898R [28]. In single channel experiments, the RyR1-D4899Q exhibits a decreased selectivity for Ca^2+^ and lower K^+^ conductance (Table 2). The experimentally observed decreased selectivity of Ca^2+^ over K^+^ in RyR1-D4899Q can be rationalized from our simulations. In simulations involving D4899Q, the ion occupancy histograms show that the peaks for K^+^ and Ca^2+^ are outside the selectivity filter compared to that of the RyR1-WT (Figure 5D). We observe that the preference for Ca^2+^ over K^+^ in the selectivity filter decreases to R = 3.1±1.6 from R = 11.3±5.6 in RyR1-WT. Further, the total ionic charge in the selectivity filter is reduced (4.3 on an average compared to 7 in RyR1-WT). RDF of ions around Q4899 (Figure 4C) strongly suggests that there is no binding of K^+^ and Ca^2+^ at the site of mutation, as also seen in Figure 5D. In simulations of RyR1-D4899Q, the RDFs and ion histogram taken together prove that Ca^2+^ and K^+^ bind only at the end of the selectivity filter especially near E4900.

Transition of an ion from a fully hydrated state to inside the channel where hydration is low is facilitated by presence of high affinity binding sites near the entrance of the channel. According to our structural model of the RyR1 pore, D4899 and E4900 residues are present at the entrance of the channel and form the first binding site of ions as they traverse from the luminal side to the cytosolic side. Hence, mutation of glutamic acid to asparagine resulting in a net loss of 4 negative charges weakens the initial binding event of cations as they enter the pore, which would result in the decrease of overall conductance of cations. Another important observation is that when just one of the two acidic residues in the selectivity filter is neutralized, Ca^2+^ binding is affected much more than K^+^, whose binding remains the same as RyR1-WT (Table 3). These results suggest the requirement of higher magnitude of negative charges in the selectivity filter to bind Ca^2+^ efficiently.

Simulations with RyR1-G4898R show a decrease in preference of Ca^2+^ in the selectivity filter (Figure 5B). The preferential occupancy ratio of Ca^2+^ over K^+^ ions (R = 4.5±0.9) is lesser than RyR1-WT (R = 11.3±5.6). The highest occupancy of Ca^2+^ ions occurs near the edge of the selectivity filter similar to that of RyR1-D4899Q, which implies an exclusion of ions from the selectivity filter. The exclusion of Ca^2+^ ions is likewise seen from the RDF of ions around D4899 in RyR1-G4898R that shows decreased affinity of Ca^2+^ to D4899 compared to wild type (Figure 4D). Thus, the introduction of a basic residue next to the acidic residues of the selectivity filter decreases ion binding in the selectivity filter, with the effect on Ca^2+^ being greater than on K^+^.

## Discussion

Elucidation of the structure-function relationship in RyR1 necessitates an atomistic model of its pore region. We constructed a model of the pore region that identifies the positions of residues critical to channel function. Furthermore, molecular dynamics simulations help us confirm the potential binding sites of ions along the pore. Considering the permeation time for different ions in RyR, our molecular dynamics simulations cannot sample statistically significant number of permeation events. However, the correlation between preferential ion occupancies seen in our simulations and experimentally measured selectivity both in RyR1-WT and its mutants suggests that preferential Ca^2+^ binding to the selectivity filter is a necessary but not a sufficient condition for selectivity. The charge space competition (CSC) model [30]–[32] provides one explanation for the selectivity for Ca^2+^ over K^+^ and Na^+^. The model attributes Ca^2+^ selectivity to the ability of Ca^2+^ (with a higher charge) to neutralize the carboxylate rich selectivity filter of Ca^2+^ channels by occupying the same space as Na^+^ (or lesser space than K^+^). The ion occupancies seen in the selectivity filter in our simulations agree well with the CSC model for RyR1 [8],[29]. An important consequence of both models is the identification of sites on the pore (4899D and 4900E) that have preferential affinity for Ca^2+^ compared to K^+^ and are experimentally shown to be sensitive to mutations.

Even though the cryoEM studies of Ludtke *et al.* [13] provide strong evidence for the structure and the orientation of helices forming the pore region, assigning the right sequence to this structure is essential for our simulations. Several biochemical and bioinformatics studies have predicted the pore-forming region of RyR1 with good agreement between each other. Balshaw *et al.* [33] first proposed that the pore forming region in RyR1 is located around 4894GGGIGD due to the striking similarity between this sequence and the selectivity filter of K^+^ channels including KcsA. Zhao *et al.* [22] performed several mutations on this highly conserved region in RyR2 and observed dramatic effects on ion conduction properties. Gao *et al.* [23] performed functional studies on similar mutants in RyR1 that also highlighted the importance of these residues in ion conduction. Using the putative selectivity filter as an anchor and the predicted positions of the membrane spanning helix from hydrophathy plots, Welch *et al.* constructed a homology model of the RyR2 pore region from KcsA [10]. In K^+^ channels, the sequence GXXXXA in the inner membrane spanning helix has been proposed to form the gating hinge [24]. The analogous glycine in RyR1 occurs in the 4934 position, in center of the inner helix predicted by hydropathy plots [34]. Studies on triadin (a transmembrane protein known to interact with RyRs [35]) indicate that three of the acidic residues of RyR1 namely, D4878, D4907 and E4908 are essential for binding of triadin to RyR1 [36]. D4878 is located in the luminal loop that connects the pore helix to the rest of the transmembrane region of RyR1. D4907 and E4908 occur in the luminal loop connecting the pore helix to inner helix and are positioned after the selectivity filter. Binding of triadin in the luminal region of RyRs inhibits channel function experimentally, which can be inferred from our model too with respect to the positions of D4907 and E4908. Thus, the sequence assignments in our model are in good agreement with results of biochemical studies.

Although the cryo-EM data for the pore-region of RyR1 is obtained from the closed state of the channel, the inner helices of RyR1 resembles that of the potassium channels in the open state (MthK). The similarity in the inner helices of RyR1 and MthK implies that the inner helices of RyR1 need not undergo major conformational change during transitions from closed to open state. The structure of the selectivity filters of the closed and open states of potassium channels (MthK and KcsA) are essentially the same, which suggests that RyR1 selectivity filter may be modeled even from its overall closed state. Moreover, the results of our simulations are in good agreement with experimental data despite modeling the pore-structure from an overall closed state of the channel.

The residues forming the selectivity filter (GGGIGDE motif) are not the only determinants for selectivity and high conductance. There are acidic residues present in the inner helix, towards the cytosolic side, D4938 and D4945 whose mutation as predicted by PNP/DFT is experimentally shown to reduce K^+^ conductance and selectivity [8],[9]. The peaks in the K^+^ and Ca^2+^ occupancies in the cytosolic vestibule are found near the positions of these residues (Figure 4). Thus there are two regions in the pore that have high affinity for ions and are known to determine selectivity. One region is present in the luminal side along the selectivity filter, while the other is present in the cytoplasmic side of the pore. The presence of negatively charged sites on either side of the channel seen in our structural model is comparable to nicotinic acetylcholine receptor ion channel [37].

RyR1-G4898R is not responsive to Ca^2+^ and caffeine and does not bind ryanodine unlike RyR1-WT which points to major altered protein conformation of the mutant channel [28]. In single channel measurements, RyR1-G4898R mutant is constitutively open to K^+^ and it loses Ca^2+^ conductance and regulation by pharmacological agents. Since we model only the pore region, the present study cannot predict the global structural changes that occur due to G4898R mutation. However, the local structural changes in the selectivity filter and the consequent loss of ion binding as seen in our simulations could account for our experimental observations on RyR1 mutants. The selectivity filters of ion channels are highly dynamic as evinced experimentally by the flickering of ion currents in single-channel measurements and structurally by the preponderance of glycines. The long, polar side chain of arginine in the selectivity filter could interact with other regions of the channel resulting in a selectivity filter that is structurally different from RyR1-WT This distortion may render the selectivity filter in a conformation that does not allow Ca^2+^ conduction. In contrast to RyR1-G4898R, RyR1-D4899Q shows Ca^2+^ and caffeine dependent Ca^2+^ release and ryanodine binding comparable to RyR1-WT [5]. Maintenance of activity suggests that changes upon mutation are localized to the selectivity filter and our simulations can identify these changes.

Our simulations identify *ab initio* the preferential localization of Ca^2+^ ions near the side chain carboxyl groups of D4899, E4900, D4938 and D4945. This result is achieved without any prior knowledge of the ion positions with respect to the selectivity filter since the initial positions of ions in the simulations are all random. Without prior bias, we are able to reproduce Ca^2+^ occupancies up to 11.3 times higher than that of K^+^, which supports the accuracy of the model of a Ca^2+^ selective channel. RyRs are important players in excitation-contraction coupling, which is fundamental to muscle contraction for movement and heart function. A mechanistic model of RyR1 will help us understand not only a functionally unique ion channel, but also shed light on an important physiological process.

## Materials and Methods

### Structural model for the pore-forming region

Our model is confined to the pore-forming region of the homotetrameric RyR1 channel. Both site directed mutagenesis and cryo electron microscopy (cryoEM) [13] have suggested that the RyRs have a pore architecture similar to K^+^ channels whose structure has been determined. Single particle cryo-EM studies on RyR1 detected several helix-like densities using the program SSEhunter [38],[39]. SSEhunter quantitatively identifies densities in a cryoEM map that may represent secondary structure elements and outputs the length and orientation of the secondary structures that can be unambiguously identified from the cryoEM map. This tool has been validated in many studies [40]–[42]. Two of these helices in each subunit, a long membrane spanning helix kinked in the middle (inner helix) and a short helix in the luminal side of the membrane (pore helix) face each other and form the backbone of the channel pore. At the resolution of the cryo-EM densities the side chains of the helices could not be resolved. The coordinates of the backbone atoms of the pore helix and the inner helix were obtained from the cryo-EM studies [13]. The kink in the middle of the inner helix (G4934) was proposed to be analogous to the gating hinge of MthK channel [27]. Sequence comparison indicates that 4894GGGIG motif is analogous to selectivity filter motif T[VI]GYG of K^+^ channels. Site directed mutagenesis suggests that 4899DE motif is also part of the selectivity filter of the RyRs.

The sequence used in constructing the pore region corresponds to M4879–E4948 (Swissprot ID: P11716) [13]. The amino acid sequence corresponding to M4879-A4893 is assigned to the pore helix while the sequence I4918-E4948 is assigned to the inner helix. In this sequence assignment, RyR1 has a long, 24-residue luminal loop (G4894-D4917), which is not visible in the cryo-EM reconstruction. Initial structure of the luminal loop is obtained by searching a database of loop structures found in SYBYL (Tripos, CA). To remove steric clashes between the loop and the helices, we further refine the loop conformation using the MODLOOP server [43], which predicts the loop conformations by satisfying spatial restraints. Using constrained all atom discrete molecular dynamics (DMD) [44], we perform simulations on the whole pore-forming region to constrain the distance between carboxyl oxygens of D4899 in the opposite monomers around 7 Å [25]. Finally, we optimize the rotamer states of all side chains using MEDUSA [45], a molecular modeling and design toolkit. Thus, we have directly used the structure of the helices identified by SSEhunter and the loop structure created using molecular modeling and low-resolution experimental constraints to create the final model of the pore structure of RyR1.

### Molecular dynamics simulations

We perform molecular dynamics simulations using GROMACS [46],[47] with the OPLSAA force field [48] modified with additional parameters for lipids [49] and ion parameters of Aqvist [50]. The RyR1 pore is placed in a pre-equilibrated DPPC bilayer with explicit solvation using genbox program in GROMACS, which removes all the water and DPPC molecules that have clashes with the pore as it is placed in the bilayer. We use the SPC model for water [51]. The simulation system shown in Figure S2A consists of the pore-forming tetramer, 405 DPPC molecules, ∼14000 water molecules and ions to make a neutral system. The concentrations of K^+^ and Ca^2+^ used in different simulations are shown in Table 1. Berendsen weak temperature coupling [52] is used with a relaxation constant of 0.5 ps. A cut-off of 10 Å is used for Van der Waals interactions and long range electrostatics is treated with Particle Mesh Ewald [53] with a grid-spacing of 12 Å and a cutoff of 10 Å.

We perform the simulations at constant volume, with a vacuum of 2 nm thickness at the top and bottom of the simulation box, to maintain asymmetry of ion concentrations at the cytoplasmic and luminal side even in the presence of periodic boundary conditions [54]. In order to ensure that the introduction of a 2 nm slab of vacuum above and below our simulation system will not affect our results, we perform one set of simulations of RyR1-WT with KCl and CaCl_2_ by replacing the 2 nm slabs of vacuum with water molecules. We calculate the ion occupancy ratios and the histogram of ion occupation along the channel axis (Table S1 and Figure S3). These results do not change the primary conclusions of this study. We observe a preferential occupation of Ca^2+^ over K^+^ in the selectivity filter with a ratio of 6.9 (within 1 SD of simulations with the vacuum slabs). However, due to the periodic boundary conditions, replacing the vacuum slab with water eliminates the partition between the cytosolic and luminal compartments, which then removes the partition between what was originally two compartments, and hence the concentration gradient.

Since the pore forming region is a small part of the entire membrane-spanning domain, the dynamics of the helices would depend on interactions with other regions of the membrane-spanning domain, which is not included in the present model. Hence, we focus on studying the interactions of the ions with the luminal loop and also the dynamics of the selectivity filter and the luminal loop. Therefore, harmonic restraints with force constants of 1000 kJmol^−1^ nm^−2^ are imposed on the C, Cα, N, and O atoms of the pore lining helices and the pore forming helices during the simulations. In the complete channel, the pore forming helix may not be in direct contact with the membrane, but to ensure minimal contact of water with the membrane spanning, pore forming helix, we surround the pore forming helix with lipids. Applying harmonic constraints on pore forming region can have dramatic effects on observed ion binding events [55]. However, in our simulations, the harmonic restraints is placed only on the pore helix and the inner helix, while the luminal loop (containing the selectivity filter) is unrestrained. The narrowest region of the pore is formed by the luminal loop, which is flexible in our simulations. The cytoplasmic side of the pore, like MthK has a much bigger volume and we predict the movement of the helix to be minimal in the time scale of our simulations and hence potential artifacts on ion binding due to the restraints on the protein should be minimal.

### Simulation methodology

The simulation system is first subjected to 1000 steps of steepest descent energy minimization. We then perform an equilibration run for 5 ns where the protein is restrained, while the lipids, water molecules and the ions are allowed to equilibrate around the protein. A 15 (or 25) ns production run is initiated after the equilibration run. The trajectory used for analysis corresponds to 5–15 (or 5–25) ns of the production run. We carry out simulations in many ionic conditions, namely CaCl_2_, KCl, NaCl, CaCl_2_+KCl, NaCl+KCl. The concentrations of ions used are shown in Table 1. Simulations are also performed on two pore mutants: RyR1-G4898R, RyR1-D4899Q.

### Analysis

We calculate ion occupancy as a function of z coordinate (along the axis of the pore) by counting the number of ions in the shaded region shown in Figure S1B. We calculate the histogram of ion occupancy with a bin width of 1 Å, which provides a picture of the ion binding sites and ion occupancies along the axis of the channel. To quantify the preferential localization of the ions, we calculate the ratio of number of each type of ion in the selectivity filter, while accounting for the differential concentration of each ionic species. We denote this ratio as R:
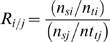
where *n_si_* is the number of ions of type *i* present in the selectivity filter, *n_ti_* is the total number of ions of type *i* present in the luminal side. Since there are no ion translocation events during our simulations, *n_t_* remains constant throughout the simulation time. *n_s_* is calculated from the area under the curves shown in Figures 3 and 5, for each corresponding simulation.

To determine the specific binding locations of the ions, we calculate radial distribution functions (RDFs) of the ions around the side chain carboxyl oxygens of D4899 and E4900 of the selectivity filter.

To confirm that the system is equilibrated, we perform our analysis on shorter stretches (5 ns) of our trajectories and them with analysis performed over the whole length of the trajectories. The ion-occupancy histograms and the RDFs of the shorter stretches of the trajectory are similar to the longer complete trajectory, which confirms that the simulations are well equilibrated (data not shown).

### Single channel recordings

Single channel measurements are performed using planar lipid bilayer method [5]. Proteoliposomes containing the purified recombinant RyR1s are added to the *cis* (SR cytosolic side) chamber of a bilayer apparatus and fused in the presence of an osmotic gradient (250 mM cis KCl/20 mM trans KCl in 20 mM KHepes, pH 7.4, 2 µM Ca^2+^). After the appearance of channel activity, trans (SR lumenal) KCl concentration is increased to 250 mM. A strong dependence of single channel activities on cis Ca^2+^ concentration indicates that the large cytosolic “foot” region faces the cis chamber of the bilayers. The trans side of the bilayer is defined as ground. Electrical signals are filtered at 2 kHz (0.5 kHz for Ca^2+^ currents at 0 mV), digitized at 10 kHz, and analyzed as described [5].

## Supporting Information

Figure S1 Histogram of ion occupancies along the axis of the channel. The distribution of ion occupancies along the axis of the channel is shown for simulation of RyR1-WT with 250 mM KCl and 70 mM NaCl. The region corresponding to selectivity filter is shaded in the plots. This region was determined from the distribution of z coordinates of Cα of G4894 and the carboxyl group of E4900 from each of the simulations.
(0.28 MB TIF)

Figure S2 The simulation system. (A) The simulation system consisting of the pore-forming tetramer (in blue), 405 DPPC molecules (in purple), ∼14000 water molecules (in red) and ions to make a neutral system with the concentration of K^+^ and Ca^2+^ fixed according to the simulation. Only two monomers are shown here for clarity. (B) Schematic for calculating ion occupancy as a function of the z co-ordinate (along the axis of the pore). We count the number of ions inside the pore (defined as the shaded region shown in the figure) as a function of the z co-ordinate and average it over the whole trajectory. This figure was created using PyMOL (http://pymol.sourceforge.net/).
(5.96 MB TIF)

Figure S3 Histogram of ion occupancies along the axis of the channel from simulations with and without vacuum slabs. RyR1-WT in 250 mM KCl and 70 mM CaCl_2_. The shaded region in the plot corresponds to the selectivity filter and this region was determined from the distribution of z component of the atomic coordinates of Cα of G4894 and the carboxyl oxygen of E4900 from the simulation.
(0.75 MB TIF)

Table S1 Ion occupancy in the selectivity filter from simulations with and without vacuum slabs.
(0.01 MB PDF)
