# Supplementary figures and images for: A Structural Model of the Pore-Forming Region of the Skeletal Muscle Ryanodine Receptor (RyR1)
Source: PLoS Comput Biol. 2009 Apr 24;5(4):e1000367. doi: 10.1371/journal.pcbi.1000367 (PMC2668181; doi:10.1371/journal.pcbi.1000367)

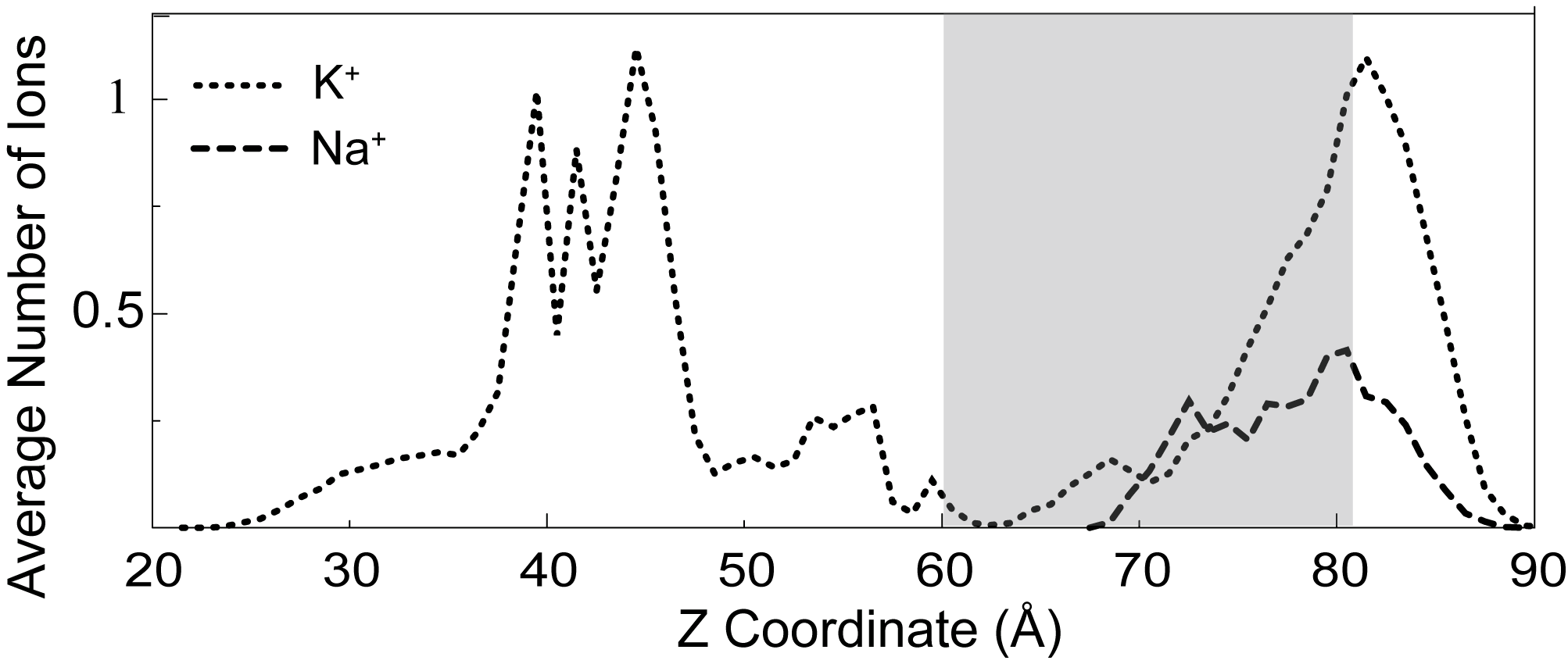

Supplement: Figure S1 — Histogram of ion occupancies along the axis of the channel. The distribution of ion occupancies along the axis of the channel is shown for simulation of RyR1-WT with 250 mM KCl and 70 mM NaCl. The region corresponding to selectivity filter is shaded in the plots. This region was determined from the distribution of z coordinates of Cα of G4894 and the carboxyl group of E4900 from each of the simulations. (0.28 MB TIF) [file pcbi.1000367.s001.tif]

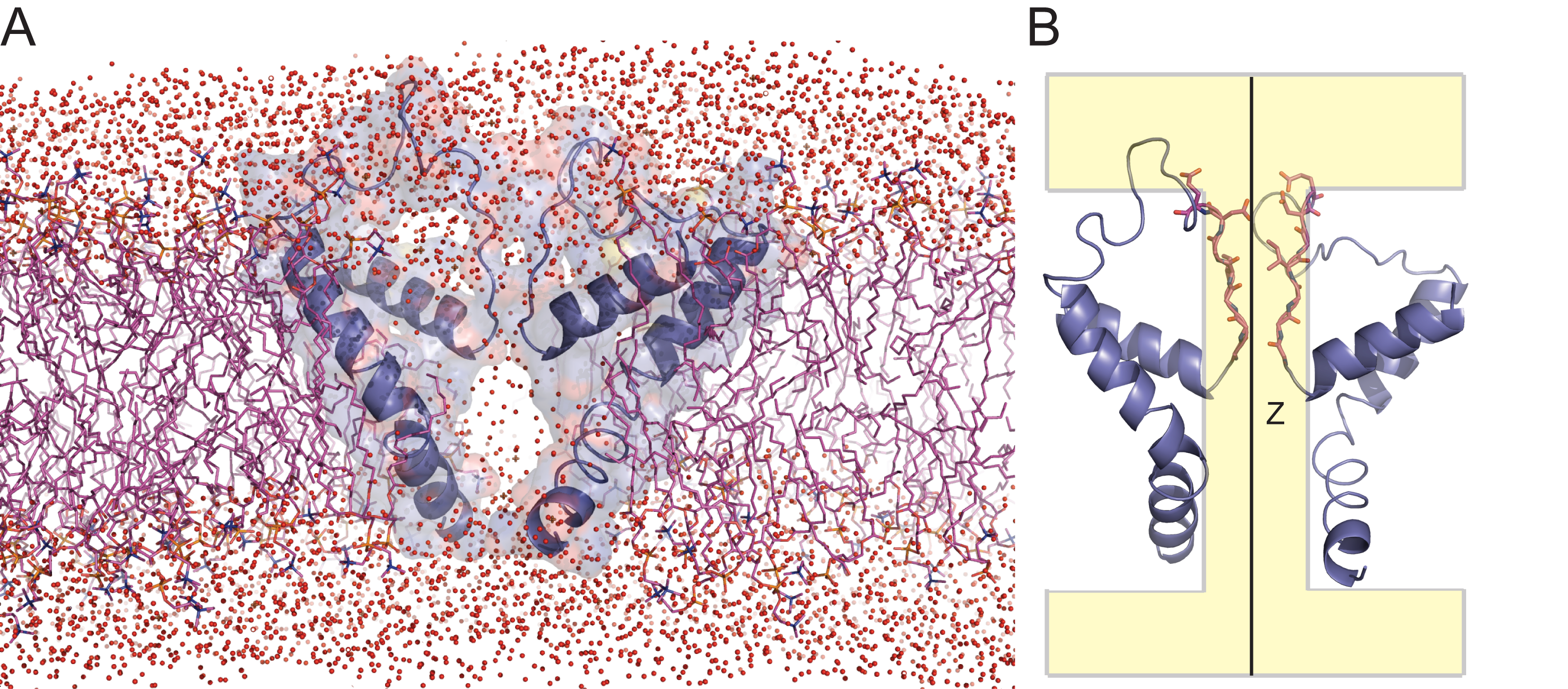

Supplement: Figure S2 — The simulation system. (A) The simulation system consisting of the pore-forming tetramer (in blue), 405 DPPC molecules (in purple), ∼14000 water molecules (in red) and ions to make a neutral system with the concentration of K+ and Ca2+ fixed according to the simulation. Only two monomers are shown here for clarity. (B) Schematic for calculating ion occupancy as a function of the z co-ordinate (along the axis of the pore). We count the number of ions inside the pore (defined as the shaded region shown in the figure) as a function of the z co-ordinate and average it over the whole trajectory. This figure was created using PyMOL (http://pymol.sourceforge.net/). (5.96 MB TIF) [file pcbi.1000367.s002.tif]

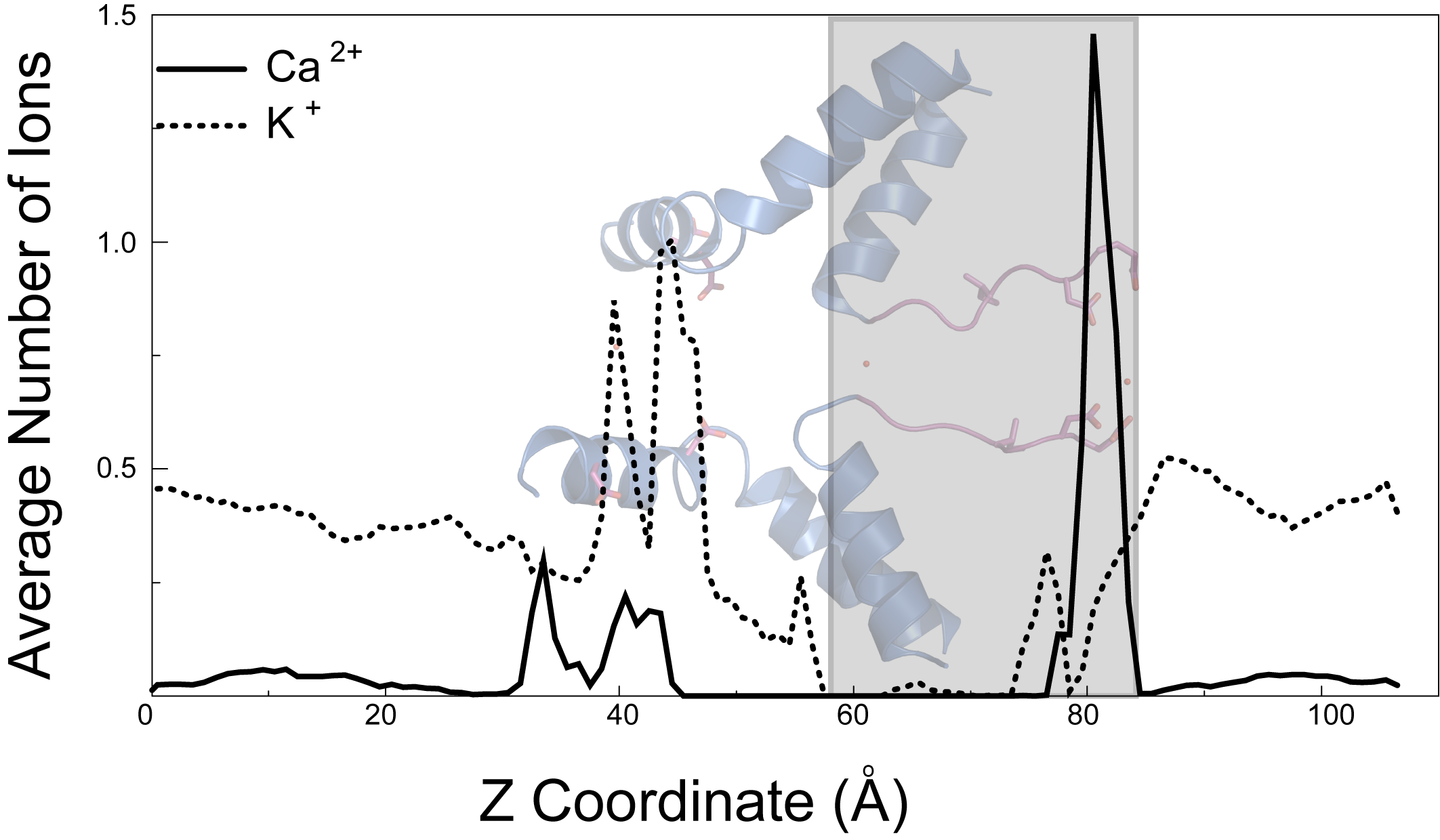

Supplement: Figure S3 — Histogram of ion occupancies along the axis of the channel from simulations with and without vacuum slabs. RyR1-WT in 250 mM KCl and 70 mM CaCl2. The shaded region in the plot corresponds to the selectivity filter and this region was determined from the distribution of z component of the atomic coordinates of Cα of G4894 and the carboxyl oxygen of E4900 from the simulation. (0.75 MB TIF) [file pcbi.1000367.s003.tif]
