# Supplementary material for: A Structural Model of the Pore-Forming Region of the Skeletal Muscle Ryanodine Receptor (RyR1)
Source: PLoS Comput Biol. 2009 Apr 24;5(4):e1000367. doi: 10.1371/journal.pcbi.1000367 (PMC2668181; doi:10.1371/journal.pcbi.1000367)
Supplement: Table S1 — Ion occupancy in the selectivity filter from simulations with and without vacuum slabs. (0.01 MB PDF) [file pcbi.1000367.s004.pdf]

Simulation	Number of ions[1]		Total number of ions in the selectivity filter	$R^‡$
	Ca^{2+}	K^+		
RyR1-WT $CaCl_2$/KCl[2]	3.4	1.7	5.1	6.9
RyR1-WT $CaCl_2$/KCl[3]	2.94 ± 0.45	1.13 ± 0.58	4.07 ± 0.95	11.31 ± 5.63

[1]In the selectivity filter, which is the shaded region shown in Figure 3 and 5. [‡]Ratio of preferential occupancy of ion1 over ion2 normalized by total number of ions present in each species. [2]Simulation with no vacuum above and below the simulation system. [3]Simulations with 2 nm vacuum above and below the simulation system.
